# Plasticity in above- and belowground resource acquisition traits in response to single and multiple environmental factors in three tree species

**DOI:** 10.1002/ece3.520

**Published:** 2013-03-07

**Authors:** Grégoire T Freschet, Peter J Bellingham, Philip O'B Lyver, Karen I Bonner, David A Wardle

**Affiliations:** 1 Department of Forest Ecology and Management, Swedish University of Agricultural SciencesUmeå, 901 83, Sweden; 2 Landcare ResearchPO Box 40, Lincoln, New Zealand

**Keywords:** Intraspecific variation, light availability, plant functional traits, plant physiological ecology, seabird burrowing, soil nutrients, specific leaf area, specific root length

## Abstract

Functional trait plasticity is a major component of plant adjustment to environmental stresses. Here, we explore how multiple local environmental gradients in resources required by plants (light, water, and nutrients) and soil disturbance together influence the direction and amplitude of intraspecific changes in leaf and fine root traits that facilitate capture of these resources. We measured population-level analogous above- and belowground traits related to resource acquisition, i.e. “specific leaf area”–“specific root length” (SLA–SRL), and leaf and root N, P, and dry matter content (DMC), on three dominant understory tree species with contrasting carbon and nutrient economics across 15 plots in a temperate forest influenced by burrowing seabirds. We observed similar responses of the three species to the same single environmental influences, but partially species-specific responses to combinations of influences. The strength of intraspecific above- and belowground trait responses appeared unrelated to species resource acquisition strategy. Finally, most analogous leaf and root traits (SLA vs. SRL, and leaf versus root P and DMC) were controlled by contrasting environmental influences. The decoupled responses of above- and belowground traits to these multiple environmental factors together with partially species-specific adjustments suggest complex responses of plant communities to environmental changes, and potentially contrasting feedbacks of plant traits with ecosystem properties. We demonstrate that despite the growing evidence for broadly consistent resource-acquisition strategies at the whole plant level among species, plants also show partially decoupled, finely tuned strategies between above- and belowground parts at the intraspecific level in response to their environment. This decoupling within species suggests a need for many species-centred ecological theories on how plants respond to their environments (e.g. competitive/stress-tolerant/ruderal and response-effect trait frameworks) to be adapted to account for distinct plant-environment interactions among distinct individuals of the same species and parts of the same individual.

## Introduction

All plants obey the same trade-offs that limit investments of the resources they acquire to different parts of cells, different tissues, and different organs (Herms and Mattson 1992). As a consequence of these and other constraints, there are broadly consistent interspecific patterns of resource allocation between plant organs, in terms of total biomass (Enquist and Niklas 2002) and in cell and tissue structure and chemistry (Kerkhoff et al. 2006; Freschet et al. 2010). Nevertheless, environmental conditions have potentially strong impacts on quantitative (Poorter et al. 2012) and qualitative (Liu et al. 2010) patterns of allocation, by driving local selection in plant genotypes and adjustments through plant phenotypic plasticity. At the species level, the relative availability of different resources can trigger adaptive and non-adaptive physiological changes in plants, which can mitigate the constraints imposed by the most limiting resources (Chapin et al. 1987; Van Kleunen and Fischer 2005; Valladares et al. 2007). Plants can therefore adjust biomass allocation to roots or shoots (i.e. quantitative adjustment) according to whether the most limiting resource is above- or belowground (Shipley and Meziane 2002; Poorter et al. 2012). Plants can also adjust the morphology and efficiency of their tissues (i.e. qualitative adjustment) to increase uptake of the most limiting resource (Ryser and Eek 2000; Hill et al. 2006) to the extent that these adjustments might be more important than changes in total mass allocation as a response to abiotic stresses (Poorter et al. 2012).

Qualitative intraspecific adjustments to abiotic stresses are widespread among plants, and are manifest in many plant traits (Chapin 1991). Aboveground, decreasing light availability commonly drives an increase in leaf area per unit mass (specific leaf area, SLA; Rijkers et al. 2000; Evans and Poorter 2001), i.e. there is an increase in the area of light capture for a constant amount of resource invested. Also, decreasing partial pressure in CO_2_ leads to higher leaf nitrogen concentrations (LNC), which is indicative of the leaf carboxylation capacity (i.e. concentration of CO_2_-fixing protein Rubisco in leaf) per unit mass invested (Ellsworth et al. 2004). Belowground, increasing nutrient limitation generally drives intraspecific increases in specific root length (SRL) (Hill et al. 2006; Ostonen et al. 2007), which represents the length of root potentially able to explore soil per unit mass invested. Together these examples illustrate how changes in the availability of any one resource, either above- or belowground, trigger changes in functional traits that determine the ability of plants to acquire that resource. However, several other environmental factors can also influence these responses (see Poorter et al. 2009 for an overview of factors influencing SLA). Under natural conditions, plants are exposed to multiple stresses and compete both for above- and belowground resources simultaneously. Furthermore, the acquisition of any one resource (e.g. carbon) generally relies on several others (e.g. light, water, and nutrients). For instance, the effect of light stress on SLA can shift from null to positive along a gradient of nitrogen availability (Meziane and Shipley 1999). Therefore, multiple environmental stresses can drive complex integration of responses at the plant level (Chapin 1991; de Kroon et al. 2005), which are potentially accountable for distinct responses across plant species with contrasting physiologies.

An important component of plant responses to the environment, other than the direction of intraspecific trait adjustments, is the strength of the response (Valladares et al. 2006). This magnitude of response can be quantified as changes in plant biomass, seed production, or any plant trait related to fitness. A high magnitude of response in those traits that determine fitness can potentially help a species maintain fitness in stressful environments and/or increase fitness in favorable environments (Richards et al. 2006). However, strong responses also come with costs and limits to fitness, such as the resources needed to generate responses, intrinsic genetic costs owing to pleiotropy, gene linkages and epistasis, or unstable plant development when environmental signals are unreliable (DeWitt et al. 1998; Van Kleunen and Fischer 2005). Although the adaptive role of intraspecific plant responses has yet to be generally demonstrated, it is likely to play an important role in determining plant fitness (Sultan 2000; Valladares et al. 2006). Several hypotheses have been proposed regarding its connections to various aspects of plant strategies (see Lavorel et al. 2009). In particular, resource-acquisitive species have been hypothesized to express a greater magnitude of intraspecific responses than resource-conservative ones because they generally appear better at exploiting varying levels of resource availability (Crick and Grime 1987; Grassein et al. 2010). However, the extent to which plant intraspecific responses are related to plant economics has received little explicit attention (Grassein et al. 2010) except in the context of plant invasions (Richards et al. 2006; Funk 2008).

In this study, we explore how multiple environmental gradients associated with light (carbon), water, and nutrient resource availability, as well as disturbance, collectively drive intraspecific changes in leaf and root traits related to the capture of these same resources. Firstly, we hypothesize that three tree species competing for the same spatial niche, but with contrasting carbon and nutrient economics, will have broadly similar responses to any single environmental factor, but will have stronger and more specific responses to combinations of several environmental factors. This is because the response of each species results from a complex integration of environmental factors at the whole-plant level (Chapin 1991; de Kroon et al. 2005). Secondly, we hypothesize that the magnitude of intraspecific responses of these species, both above- and belowground, will be related to the extent to which their economies are acquisitive or conservative. Thirdly, we hypothesize that, despite the potentially widespread coordination in leaf and root economics across species (Reich et al. 2008; Liu et al. 2010), there will be uncoordinated shifts in nutrient and carbon economics above- versus belowground within species, because leaf and root traits (associated with the acquisition of above- and belowground resources respectively) should respond differently to (orthogonally) varying levels of resources above- versus belowground. We test these hypotheses by measuring a set of above- and belowground plant traits representative of plant carbon and nutrient economics on three tree species at the population level within the same ecosystem along strong gradients of light, soil nutrient concentrations, moisture, and disturbance.

## Materials and Methods

### Study system

Korapuki is a small, isolated, forested 18 ha island of volcanic origin and relatively steep terrain in the Mercury Islands, off the NE coast of the North Island of New Zealand (36°39′S, 175°50′E, maximum elevation 81 m). The climate is warm temperate with mean monthly temperatures ranging from 14°C in June to 19.5°C in January and annual precipitation of *c*. 2000 mm. The vegetation cover is generally dense with a tall canopy of *Metrosideros excelsa* (Myrtaceae) over a sub-canopy of *Coprosma macrocarpa* (Rubiaceae), *Melicytus ramiflorus* (Violaceae), and *Pittosporum crassifolium* (Pittosporaceae) – hereafter *Coprosma*, *Melicytus,* and *Pittosporum* – which is typical of vegetation on warm temperate islands in New Zealand (Atkinson 2004). A variety of burrowing seabirds (primarily *Pterodroma macroptera gouldi*, but also *Pelecanoides urinatrix*, *Pterodroma pycrofti*, *Puffinus assimilis haurakiensis,* and *Puffinus gavia*, all Procellariiformes) breed on the island, some of which have become more abundant after the eradication of introduced rabbits (*Orcytolagus cuniculus*) and Pacific rats (*Rattus exulans*) in 1986–1987 (Towns 2002). Burrowing by these seabirds causes considerable soil disturbance (Fukami et al. 2006), and as a consequence, on this island ground cover ranges from almost bare soil in heavily burrowed areas to light understorey vegetation and abundant leaf litter in areas with little seabird influence.

Square plots of 10 × 10 m were randomly assigned to the island using ArcGIS 10 software (ESRI, Redlands, CA, USA) from which fifteen plots representing the whole range of seabird burrowing activity (from 0 to 94 burrows per plot) were selected. Our plot selection excluded areas of the island such as rocky shoreline or those that were inaccessible because of topographical constraints. The distance between plots varied between *c*. 50 and 600 m. The three dominant sub-canopy tree species, *Coprosma*, *Melicytus* and *Pittosporum* were present on all plots – but only as mature trees in 14, 12, and 15 plots respectively – and were therefore the focus of this study. The relatively homogeneous cover of these three species across the island creates an ideal system for studying the impact of spatial environmental variations on plant plasticity. Selection of tree species that grow to a similar size has the advantage of limiting potential differences in species responses related to plant size (e.g. the capacity of plants to access heterogeneously distributed patches of light or soil resources; Casper and Jackson 1997). Nevertheless, these three species have contrasting carbon and nutrient economics, as evidenced by contrasting values of leaf and root traits representative of plant economics (see Wardle et al. 2009; this study), and therefore potentially different capacities to overcome resource stresses and disturbances, i.e. potentially species-specific responses. Furthermore, Korapuki is characterized by strong belowground spatial heterogeneity and variation in upper canopy structure over even small spatial scales, making it ideal for testing questions about spatial resource heterogeneity. The presence of seabirds is likely to create strong gradients of disturbances and soil fertility across the island (Bancroft et al. 2005; Mulder et al. 2011) and the occurrence of gaps in the upper canopy leads to considerable spatial variation in the light transmittance to the sub-canopy species. Finally, all sampled tree populations on Korapuki (which is small and isolated) have presumably regrown from the same tree population which has developed since the 1950s after earlier deforestation of the island (Towns 2002). Although new genetic material may have been brought in from distant islands, most passerines involved in the dispersal of fruits from the focal trees in our study disperse most seeds over short distances (tens of meters, Stansbury 2001; Williams 2006). Therefore, differences between tree populations that result from genotypic adaptation to local conditions on the island are presumably very small, and the trait variation reported here is therefore assumed almost entirely to reflect phenotypic plant responses to the environment.

### Environmental measures

In July 2010, the number of seabird burrow entrances and eggs (one per breeding pair) were counted per plot. These two variables were strongly positively related across plots (*R*^2^ = 0.87; *P* < 0.001) and we therefore used only burrow density as a measure of seabird impact. The density of burrows per area is a relative measure both of the nutrient transfer to the soil from the seabirds and disturbance by the seabirds to plant roots resulting from excavation and upkeep of the burrows (Roberts et al. 2007; Wardle et al. 2009). Increasing burrow density is also likely to have some impact on the water content and compaction of soils by favoring water drainage and aeration (Bancroft et al. 2005).

Canopy openness was estimated from two hemispherical photographs per plot (*c*. 1.3 m height) using Gap Light Analyzer 2.0 software (Simon Fraser University, Burnaby, Canada). It therefore represents an index of light availability to leaves of the lower sub-canopy (on average from 2 to 4 m in height), including those of our three focal tree species.

Four 10 × 10 × 10 cm soil pits were sampled per plot in March 2010, pooled, homogenized and used for further soil analysis. The mass and volume of this soil was used to calculate bulk density. Soil moisture content was measured on a sub-sample dried to constant weight at 60°C. Since weather was warm and dry with no precipitation during the entire sampling period, soil moisture content was used here as an index of water availability for plants. The two main form of N available for plants, i.e. NO_3_-N and NH_4_-N, were extracted with KCl solution and measured by auto-analyser (Lachat Instruments Division, Zellweger Analytics Inc., Milwaukee, WI, USA). A measure of available phosphorus for plants, Olsen-P, was measured using sodium bicarbonate digest.

### Plant traits

In November 2011, *Coprosma*, *Melicytus* and *Pittosporum* were sampled from each plot for shade leaves and fine superficial roots sampled whenever possible from a minimum of ten distinct plant individuals. Fifty healthy, fully expanded leaves (presumably from the current year) were sampled per species per plot from the inside of the sub-canopy (between *c*. 2–3 m in height). Ten to twenty roots per species per plot were tracked down from the tree stem to the fine root tips and dug out. All leaf and root material was kept cool and wet until cleaning and further processing in the lab. All root branches below 1 mm in diameter, except explorative roots, were clipped off, thoroughly cleaned from residual soil and taken as “fine roots.” For each species and plot, all 50 leaves and all fine root material was used to estimate specific leaf area (SLA, mm^2^/mg) and specific root length (SRL, m/mg) as well as leaf and fine root dry matter content (DMC, fresh to dry weight ratio, mg/g). Measurements followed Cornelissen et al. (2003). All leaves and fine roots were then coarsely ground and homogenized manually before taking a sub-sample for total N and P concentration analysis using acid digest and colorimetric methods of Blakemore et al. (1987).

### Data analysis

The single and multiple influences of four environmental parameters (light availability, soil nutrient availability, soil moisture availability, and seabird burrow density) on intraspecific variation in leaf and root functional traits (SLA–SRL, DMC, N and P) were assessed separately for each plant species using simple and multiple linear regressions. In order to maximize the statistical power of our tests and minimize multicollinearity, two highly correlated environmental parameters were transformed into one summarizing index using a principal component analysis (PCA): “soil nutrient availability” was thus estimated as the first axis of a PCA that summarizes 89% of variation in available N (nitrate + ammonium) and available P (Olsen-P). Seabird burrowing activity was included in multiple linear regressions because it represents the impact of disturbances caused by seabird burrowing activity in addition to the effects of seabirds on soil nutrients, so could potentially explain variability that ‘soil nutrient availability’ cannot. Seabird burrow density was log_10_-transformed for all single and multiple regressions concerned to comply with normality assumptions. For each species × trait combination, the most relevant models were identified using Akaike's Information Criterion (AIC). Models displaying AIC ≤ lowest AIC + 1 were considered equivalent. Results of regressions with four parameters were not shown because of the low number of observations per parameter and because their AIC never fell below these with less parameters.

To illustrate the effect of multiple contrasting environmental influences on the intraspecific variation in whole-plant economic strategies of our three tree species, we performed a PCA on all leaf and root traits of all species simultaneously. We used *t*-tests among pairs of species to determine whether the ranking of these three species along the first axis of the PCA, which represented a “plant economics spectrum” (Freschet et al. 2010), changed across environmental influences (i.e. across plots).

In order to compare the magnitude of plastic responses among tree species and organs to the same environmental variation, we focused on intraspecific trait responses to multiple environmental influences rather than to single environmental factor or sets of environmental factors (Valladares et al. 2006). This is because intraspecific shifts in plant trait values were generally co-determined by several environmental parameters which often differed across species. Consequently, measuring trait responses of different species to the same set of (subjectively chosen) environmental factors would make little sense in this context. Specifically, for each trait × species combination, we transformed trait values at each plot into percentages of deviation from the mean trait value across all plots. Then, for each trait, we used two-sample Fisher's *F*-tests to test for differences between species in the homogeneity of variance in these percentages of deviation from the mean trait value. For each species, we also used Fisher's *F*-tests in the same way to assess the homogeneity of intraspecific variance between functionally analogous leaf and root traits (i.e. SLA vs. SRL; root vs. leaf DMC, N and P). Expressing the magnitude of intraspecific trait responses as a relative deviation from the species mean trait value is equivalent to using coefficients of variation (CV) and allows comparisons of species' variance independently of the mean plant trait value.

Standardized major axes regressions were used to test the coordination between functionally equivalent leaf and root traits across the 15 plots for each species and, when significant, slope comparison procedures were performed between species (SMATR-package; Warton et al. 2006).

## Results

Among all possible pairs of four environmental parameters (see Table 1 for an outline of parameters' ranges, means, and variabilities), only soil nutrient availability and seabird burrow density were significantly positively related (*R*^2^ = 0.45; *P* < 0.01). Light availability was largely unrelated to soil moisture content, soil nutrient availability, and seabird disturbance (*R*^2^ = 0.18, 0.10, 0.20, respectively; *P* ≥ 0.10). Soil moisture content was unrelated to seabird disturbance (*R*^2^ = 0.11; *P* ≥ 0.10) and only marginally negatively related to soil nutrient availability (*R*^2^ = 0.25; *P* = 0.06).

**Table 1:** Range of the main environmental gradients on Korapuki Island

Environmental parameters	Range	Mean	(±SD)
Light availability (% canopy openness)	1.4−11.2	6	(±2.8)
Soil NO_3_-N and NH_4_-N (mg/kg)	6.5−173.6	52	(±51)
Soil Olsen-P (mg/kg)	10.0−994.0	273	(±254)
Soil moisture content (% dry weight)	127−267	177	(±43)
Seabird burrow density (m^−2^)	0−0.94	0.25	(±0.26)

### Multiple environmental factors co-determine intraspecific trait responses

**Figure 1:**
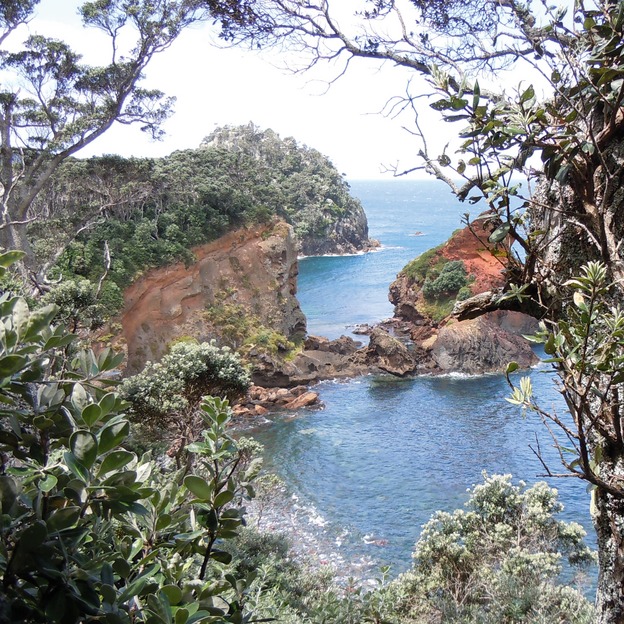
South coast of Korapuki. Photo by G.T. Freschet.

The PCA primary axis scores for the three species with regard to their traits differed greatly, although there was some overlap between *Coprosma* and each of the other two species along the plant economics spectrum axis (Fig. 1). The species' ranking along this axis was strictly conserved across the 15 plots, indicating that there were broadly consistent influences of environmental parameters on multiple traits of the three distinct tree species.

More specifically, several models that included either single or multiple environmental factors often explained more than half of the total trait variance across the plots for all three species (Tables 2 and 3; Tables S1 and S2). For most plant traits, although we observed largely consistent trends across all three tree species, the best models often differed at least slightly between species. This was particularly true for SLA, SRL, leaf and root N concentrations, root DMC and root P concentration, but less so for leaf DMC and leaf P concentration.

**Table 2:** Linear models of single and multiple environmental parameters' fit of specific leaf area and specific root length of three sub-canopy tree species

	Specific leaf area (mm^2^/mg)									Specific root length (mg)								
		
	*Pittosporum*			*Coprosma*			*Melicytus*			*Pittosporum*			*Coprosma*			*Melicytus*		
						
	Sign	*R*^2^	AIC	Sign	*R*^2^	AIC	Sign	*R*^2^	AIC	Sign	*R*^2^	AIC	Sign	*R*^2^	AIC	Sign	*R*^2^	AIC
*Burrow* density	**–**	0.45	1.3	**−**	**0.44**	**21.2**	**−**	**0.56**	**20.4**	+	0.28	43.9	+	0.19	68.5	+	0.03	42.4
Soil *Nutrient* availability	**−**	0.40	2.7	**−**	0.10	27.7	**−**	0.10	29.1	+	**0.49**	**38.7**	+	0.53	61.0	+	**0.42**	**36.8**
Soil *Water* content	+	0.58	−2.8	+	0.19	26.2	+	0.27	26.5	**−**	0.02	48.5	**−**	0.48	62.4	**−**	0.06	42.1
*Light* availability	**−**	0.52	−0.7	**−**	0.33	23.6	**−**	0.38	24.6	**−**	0.00	48.7	+	0.11	69.9	+	0.11	41.5
Burrow + Nutrient	−,−	0.51	1.7	−, +	0.46	22.6	−, +	0.59	21.8	+, +	0.50	40.5	−, +	0.53	63.0	−, +	**0.50**	**37.2**
Burrow + Water	−, +	0.77	−9.9	−, +	**0.50**	**21.5**	−, +	**0.63**	**20.6**	+, +	0.28	45.9	+, −	0.54	62.7	+, −	0.06	44.1
Burrow + Light	−,−	0.67	−4.4	−,−	**0.53**	**20.5**	−,−	**0.62**	**20.7**	+, −	0.38	43.6	+, +	0.22	70.1	−, +	0.11	43.5
Nutrient + Water	−, +	0.66	−4.1	−, +	0.21	27.9	−, +	0.27	28.5	+, +	**0.55**	**38.9**	+, −	**0.68**	**57.4**	+, +	0.45	38.2
Nutrient + Light	−,−	0.69	−5.5	−,−	0.35	25.1	−,−	0.39	26.5	+, −	**0.58**	**37.9**	+, +	0.54	62.7	+, +	0.43	38.6
Water + Light	+, −	0.77	−9.9	+, −	0.38	24.6	+, −	0.48	24.6	−,−	0.04	50.2	−, +	0.48	64.4	−, +	0.13	43.3
Burrow + Nutrient + Water	−, −, +	0.77	−7.9	−, +, +	**0.57**	**21.4**	−, +, +	**0.71**	**19.7**	+, +, +	0.55	40.7	−, +, −	0.69	59.3	−, +, +	0.50	39.1
Burrow + Nutrient + Light	−,−,−	0.72	−4.9	−, +, −	0.56	21.7	−, +, −	0.65	21.8	+, +, −	**0.61**	**38.7**	−, +, +	0.54	64.5	−, +, +	**0.59**	**37.0**
Burrow + Water + Light	−, +, −	**0.86**	**−15.5**	−, +, −	0.56	21.8	−, +, −	0.66	21.4	+, −, −	0.38	45.5	+, −, −	0.54	64.6	−, −, +	0.14	45.1
Nutrient + Water + Light	−, +, −	0.82	−11.8	−, +, −	0.38	26.5	+, +, −	0.48	26.6	+, +, −	0.60	39.1	+, −, −	0.69	59.3	+, +, +	0.47	39.8

Signs of model parameters are displayed for each model. The fraction of total trait variation explained by each model is represented by regression coefficients R^2^. Models with Akaike's information criterion (AIC) ≤ (lowest AIC + 1) were considered of high likelihood and indicated in bold. The number of tree populations is 15 for *Pittosporum crassifolium,* 14 for *Coprosma macrocarpa*, and 12 for *Melicytus ramiflorus*.

**Table 3:** Linear models of single and multiple environmental parameters' fit of leaf and fine root nitrogen concentration of three sub-canopy tree species

	Leaf nitrogen content (%)									Root nitrogen content (%)								
		
	*Pittosporum*			*Coprosma*			*Melicytus*			*Pittosporum*			*Coprosma*			*Melicytus*		
						
	Sign	*R*^2^	AIC	Sign	*R*^2^	AIC	Sign	*R*^2^	AIC	Sign	*R*^2^	AIC	Sign	*R*^2^	AIC	Sign	*R*^2^	AIC
*Burrow* density	+	0.59	−65.6	+	0.07	−43.9	+	0.01	−18.0	+	0.64	−55.1	+	0.29	−27.4	+	0.10	−23.5
Soil *Nutrient* availability	+	0.60	−65.7	+	0.36	−49.0	+	0.44	−24.8	+	0.64	−54.7	+	0.36	−28.9	+	0.53	−31.3
Soil *Water* content	**−**	0.04	−52.9	**−**	0.00	−42.9	**−**	0.04	−18.4	**−**	0.05	−40.3	**−**	0.00	−22.6	**−**	0.24	−25.5
*Light* availability	+	0.00	−52.2	**−**	0.07	−43.8	**−**	0.08	−18.9	+	0.01	−39.7	**−**	0.06	−23.4	**−**	0.01	−22.3
Burrow + Nutrient	+, +	0.71	−68.7	−, +	0.39	−47.7	−, +	0.58	−26.3	+, +	0.77	−59.3	+, +	0.40	−27.7	−, +	0.55	−29.8
Burrow + Water	+, +	0.59	−63.7	+, +	0.07	−41.9	+, −	0.04	−16.4	+, +	0.65	−53.2	+, +	0.32	−25.9	+, −	0.26	−23.8
Burrow + Light	+, −	0.74	−70.1	+, −	0.25	−44.8	+, −	0.15	−18.0	+, −	0.73	−57.1	+, −	0.58	−32.8	+, −	0.21	−23.0
Nutrient + Water	+, +	0.64	−65.3	+, +	0.43	−48.7	+, +	0.48	−23.7	+, +	0.68	−54.6	+, +	0.46	−29.1	+, −	0.55	−29.7
Nutrient + Light	+, −	0.66	−66.5	+, −	**0.58**	**−53.2**	+, −	**0.75**	**−32.3**	+, −	0.66	−53.9	+, −	0.57	−32.5	+, −	**0.69**	**−34.1**
Water + Light	−,−	0.05	−51.0	−,−	0.10	−42.3	−,−	0.18	−18.3	−, +	0.05	−38.3	−,−	0.07	−21.6	−,−	0.34	−25.1
Burrow + Nutrient + Water	+, +, +	0.75	−69.0	−, +, +	0.46	−47.4	−, +, +	0.60	−25.1	+, +, +	0.81	−60.4	+, +, +	0.49	−28.1	−, +, −	0.57	−28.3
Burrow + Nutrient + Light	+, +, −	**0.86**	**−78.1**	−, +, −	0.58	−51.2	−, +, −	0.76	−31.3	+, +, −	**0.86**	**−64.7**	+, +, −	**0.71**	**−35.8**	+, +, −	0.69	−32.1
Burrow + Water + Light	+, −, −	0.74	−68.4	+, −, −	0.26	−43.1	+, −, −	0.22	−17.0	+, −, −	0.73	−55.2	+, −, −	0.58	−30.8	+, −, −	0.44	−25.2
Nutrient + Water + Light	+, +, −	0.68	−65.2	+, +, −	0.60	−51.6	+, +, −	0.75	−30.6	+, +, −	0.69	−53.1	+, +, −	0.60	−31.4	+, −, −	**0.73**	**−33.9**

Signs of model parameters are displayed for each model. The fraction of total trait variation explained by each model is represented by regression coefficients *R*^2^. Models with Akaike's information criterion (AIC) ≤ (lowest AIC + 1) were considered of high likelihood and indicated in bold. The number of tree populations is 15 for *Pittosporum crassifolium,* 14 for *Coprosma macrocarpa,* and 12 for *Melicytus ramiflorus*.

Among the models with the lowest AIC (i.e. ≤ lowest AIC + 1), population level SLA across plots was best fitted by models using seabird disturbance, soil moisture content and light availability for *Pittosporum,* disturbance and light availability for *Coprosma*, and disturbance, soil moisture content and soil nutrient availability for *Melicytus* (Table 2). However, when single parameter predictions were considered, disturbance, light and soil moisture on their own consistently explained significant amounts of variation in SLA. While intraspecific variation in SRL was best fitted by models using disturbance, soil nutrient availability and light for both *Pittosporum* and *Melicytus*, it was best fitted by models that integrated soil nutrient availability and moisture for *Coprosma* (Table 2). Also, the sign of disturbance and light parameters of the models appeared reversed between *Pittosporum* and *Melicytus*. Thus, in all three cases only soil nutrient availability had a consistent (positive) influence on SRL. When considered alone it explained a large proportion of the total variability in SRL across plots, i.e. 49, 53, and 42% for *Pittosporum*, *Coprosma,* and *Melicytus*, respectively.

For all three species, soil nutrient availability alone explained a substantial proportion of the variability across plots in leaf and root N concentrations (Table 3). However, *Pittosporum* LNC was most strongly influenced by a combination of soil nutrient availability, light and disturbance, while LNC for *Coprosma* and *Melicytus* were most responsive to soil nutrient availability and light only. Intraspecific variability for root N concentration (RNC) was also largely driven by nutrients and light, but while *Pittosporum* and *Coprosma* responded most strongly to these in combination with disturbance, *Melicytus* responded most strongly to them in combination with soil moisture (Table 3).

Intraspecific variability in leaf dry matter content (LDMC) was relatively well fitted by models containing disturbance alone or including both disturbance and light for *Coprosma* and *Melicytus*, but no models were satisfactory for *Pittosporum* (Table S1). In contrast, while largely different models best fitted intraspecific variability in root dry matter content (RDMC) across species, soil nutrient availability emerged as a consistent driver across all three species and explained 64, 63, and 58% of the variability in RDMC of *Pittosporum*, *Coprosma,* and *Melicytus*, respectively (Table S1).

Finally, while a model that included light, soil nutrient availability and moisture explained up to 59% in the intraspecific variability in leaf P concentration (LPC) of *Melicytus*, none of the environmental parameters could adequately explain the intraspecific variability in LPC for *Pittosporum* and *Coprosma* (Table S1). In contrast, the intraspecific variation in root P concentration (RPC) across species appeared mostly driven by disturbance and soil nutrient availability although, depending on the species, either soil moisture or light appeared to slightly improve model fits (higher *R*^2^ but similar AIC).

### A broadly similar magnitude of intraspecific trait responses across organs and species

**Figure 2:**
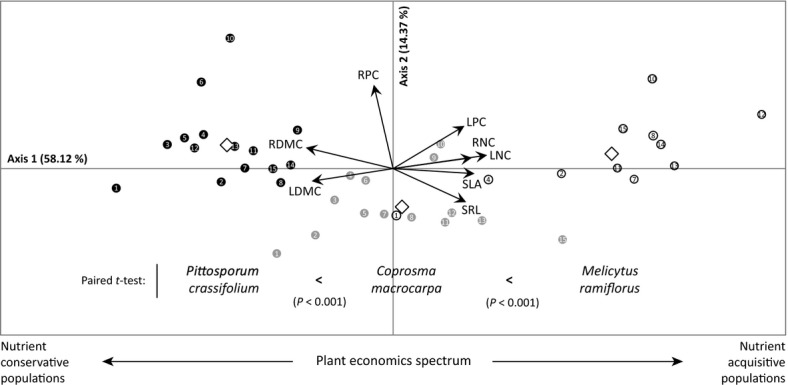
Principal component analysis (PCA) on eight leaf and root traits representative of plant nutrient and carbon economics for all tree populations (15 plots × 3 species). Plot numbers are indicated for each population of *Pittosporum crassifolium* (•), *Coprosma macrocarpa* (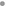), and *Melicytus ramiflorus* (○). The *x*-axis, summarizing 58% of total trait variation, ordinates tree populations from those showing the most resource-conservative traits (left) to those displaying the most resource-acquisitive ones (right). Results of paired-samples *t*-tests between the *x*-axis scores of *Coprosma* and *Pittosporum* and *Coprosma* and *Melicytus* show consistent rankings of the three tree species across all plots. Species centroids are displayed as diamonds. Plant traits: specific leaf area (SLA), specific root length (SRL), leaf and root dry matter content (LDMC, RDMC), leaf and root nitrogen (LNC, RNC) and phosphorus concentrations (LPC, RPC).

Only leaf and root DMC followed the hypothesized trend of increasing trait variance from *Pittosporum* to *Coprosma* to *Melicytus*, and the only difference in trait variance among species that was statistically significant was for LDMC between *Pittosporum* and both *Coprosma* and *Melicytus* (*P* = 0.01 in both cases; Fig. 2). Furthermore, variances of six traits, including RDMC, leaf and root N and P concentrations, and SLA, were not significantly different among the three tree species. Also, the intraspecific variance in SRL was significantly higher for *Coprosma* than for both *Pittosporum* and *Melicytus* (*P* = 0.01 in both cases).

At the species level, mostly similar variances were observed between functionally equivalent leaf and root traits, although in three instances root traits showed higher variance than their leaf trait counterparts. Thus, *Pittosporum* displayed significantly higher variance in root than leaf DMC (*P* = 0.04) and *Coprosma* showed significantly higher variance in root than leaf N concentration (*P* = 0.04) and in SRL than SLA (*P* < 0.01).

### Orthogonal responses of analogous leaf and root traits to multiple environmental influences

**Figure 3:**
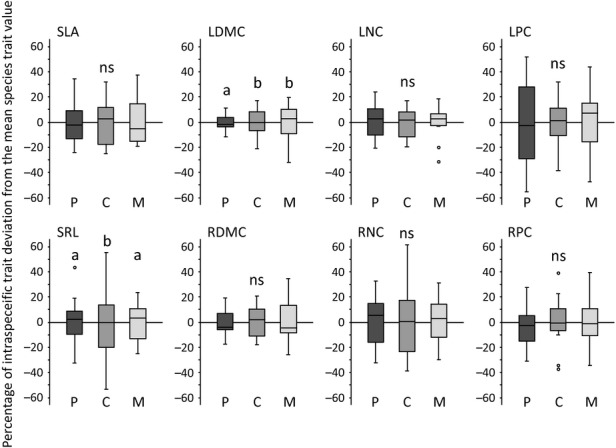
Percentage deviation of *Pittosporum crassifolium* (P), *Coprosma macrocarpa* (C), and *Melicytus ramiflorus* (M) tree populations from their respective mean species trait value, for eight above- and belowground traits. Species are ranked from left to right from the most conservative to the most acquisitive. Each boxplot represents the intraspecific trait variation of a group of 12–15 tree populations (i.e. plots) depending on species, where population trait values are expressed as percentage deviation from the mean species trait value across all populations (central line is median; mean is zero; boxes indicate the first and third quartiles; whiskers are confidence limits; empty circles are outliers). Significant (*P* < 0.05; as indicated by different letters) and non-significant (ns) heterogeneity in intraspecific trait variance were tested using two-sample Fisher's *F*-tests on all pairs of species for each trait. Plant traits: specific leaf area (SLA), specific root length (SRL), leaf and root dry matter content (LDMC, RDMC), leaf and root nitrogen (LNC, RNC) and phosphorus concentrations (LPC, RPC).

**Figure 4:**
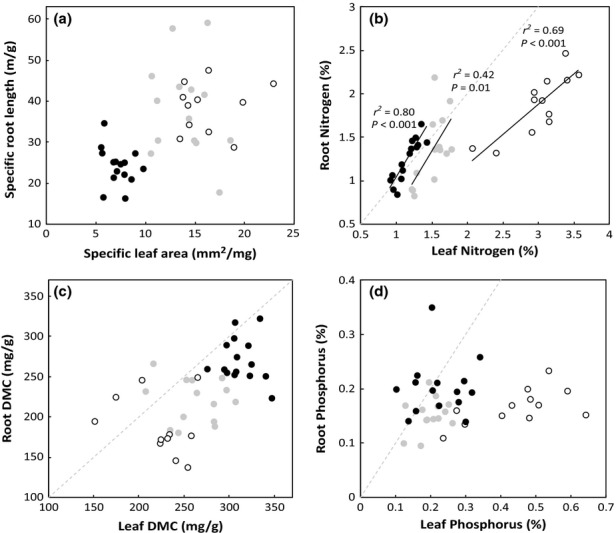
Standardized major axis (SMA) regressions between analogous leaf and root functional traits across tree populations exposed to varying environmental conditions, with points representing individual plots. Tree species are *Pittosporum crassifolium* (•), *Coprosma macrocarpa* (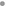), and *Melicytus ramiflorus* (○). Significant relationships (*P* < 0.05) between leaf and fine roots of the same species are indicated by their SMA regression lines, r^2^, and *P*-values. Dotted lines represent 1:1 relationships.

As described above, for each of the three species, different environmental parameters appeared to drive intraspecific shifts in analogous above- and belowground traits, such as SLA versus SRL (Table 2), LDMC versus RDMC (Table S1), and LPC versus RPC (Table S2). In contrast, LNC and RNC were driven by a similar combination of parameters, notably nutrient and light availability (Table 3). As a consequence, while intraspecific variation in LNC and RNC were significantly related (Fig. 3b), all other pairs of leaf and root traits were not (Fig. 3a, c, d). The slope of LNC–RNC relationship for *Melicytus* was significantly smaller than this for the two other species (*P* < 0.01 in both cases) Fig. 4.

## Discussion

### Multiple environmental factors often co-determine intraspecific trait responses and drive partially species-specific responses

Most traits of the three tree species showed similar responses to the same environmental factors when factors were considered separately (e.g. Wahl et al. 2001; Rozendaal et al. 2006), and, consistent with our first hypothesis, combinations of several factors often explained higher intraspecific trait variation than single factors. For instance, light availability is generally considered as the main driver of intraspecific shifts in SLA (Evans and Poorter 2001; Rozendaal et al. 2006). However, for all three tree species we observed a strong influence of soil disturbance caused by seabird burrowing and soil moisture content (see also Poorter et al. 2009), which together explained more variance than the otherwise significant influence of light availability alone. Also, even though the variance in leaf and root N concentrations explained by light availability was negligible for all three species, the combination of light availability and soil nutrient availability had a much stronger effect on leaf and fine root N concentrations than did soil nutrient availability alone. These results suggest that considering phenotypic responses to single environmental parameters, as done by all but a handful of studies, is inadequate to predict intraspecific plant trait responses to multiple co-occurring environmental factors (see also Meziane and Shipley 1999; Wahl et al. 2001; Valladares et al. 2007; Poorter et al. 2009).

Two patterns of trait response to multiple environmental factors differed substantially from those generally observed in studies of single environmental factors. First, in addition to the positive impact of soil nutrient availability on leaf and root N concentrations (Xia and Wan 2008), light availability appeared to co-determine the N concentrations of leaves and fine roots of our three sub-canopy species. Increasing leaf N concentrations with decreasing light may correspond to an adaptive investment to improve light capture capacity in situations where carbon is the limiting resource (Evans and Poorter 2001). However, this trend can be more parsimoniously explained by higher carbon assimilation and thereby plant growth in higher light conditions, which potentially creates a N sink in growing plant parts and thereby decreases the ‘luxury consumption’ of N in both leaf and fine root tissues (Van Wijk et al. 2003). Second, in contrast to most previous studies (e.g. Hill et al. 2006; Ostonen et al. 2007), we observed decreasing SRL and increasing RDMC with decreasing soil nutrient availability (but see Ryser and Lambers 1995; Yano and Kume 2005). This trend may benefit plants by increasing their root lifespan in environments that provide a slower return on investment (Eissenstat et al. 2000; Ryser 2006) as observed across species of low and high resource environments (Ryser and Eek 2000; Freschet et al. 2010). Additionally, decreasing SRL may also represent an increase in fine root mycorrhizal associations (Eissenstat et al. 2000) in conditions of high nutrient stress.

Our results also revealed some species-specific responses to spatial variation in light, soil nutrient, and moisture availabilities, as well as root-damaging disturbance by seabirds. For instance, while RDMC decreased with soil nutrient availability for all three species, nutrient and light availability strongly co-determined RDMC in *Pittosporum*, and nutrient, light, and seabird burrowing co-determined RDMC in *Melicytus*. Our results, together with previous studies exploring gradients in soil nutrient availability (Lawrence 2003), soil moisture content (Cornwell and Ackerly 2009), and temperature (Albert et al. 2010), suggest that plant communities could exhibit complex responses to changing environmental conditions (Lavorel et al. 2009), which portends substantial uncertainty in trait-based projections of plant assemblages under scenarios of global change (Thuiller et al. 2008).

### Does the strength of intraspecific plant trait responses relate to plant resource acquisition strategy?

We found broadly similar amplitudes of intraspecific plant trait responses across all three tree species to the same multiple environmental influences, irrespective of their nutrient and carbon economics (Fig. 2; see also Funk 2008). This result did not provide support for our second hypothesis. Only leaf and root DMC displayed the expected trend of increasing strength of intraspecific response (although mostly non-significant) as the species became more resource-acquisitive, which is consistent with results for leaf DMC in alpine grassland communities (Lavorel et al. 2009; Grassein et al. 2010). Our results therefore suggest that the capacity for large intraspecific variation in traits might be equally important for both acquisitive and conservative species. Environmental heterogeneity is widespread within both fertile and unfertile sites and plants characteristic of both environments may have a selective benefit through exhibiting strong intraspecific trait responses. Studies featuring a greater number of species remain necessary to answer whether the magnitude of plant intraspecific responses relates to their resource economics. Also, our study system is unlikely to cover the entire range of environmental conditions that our focal species can occupy. Therefore, the general lack of relationship observed in our study between the strength of plant intraspecific response and plant economics does not exclude the possibility that between-species differences in plasticity could be observed with respect to the physiological limits of plastic responses (rather than the proportional strength of the response).

### Multiple environmental influences drive decoupled plant resource acquisition strategies above- and belowground

In support of our third hypothesis, we observed uncoordinated shifts within species for three of four functionally analogous leaf and root traits, which resulted from contrasting responses of leaf and root traits to the same multiple environmental gradients. Growing evidence suggests that there are broadly coordinated economics of both carbon and nutrients between plant above- and belowground organs at the between-species level (Wright and Westoby 1999; Freschet et al. 2010; Liu et al. 2010), most likely as a consequence of allometric, physiological, and ontogenetic constraints in plant evolutionary selection (Ackerly et al. 2000; Reich et al. 2003b; Kerkhoff et al. 2006). However, these relationships are not always apparent (Reich et al. 2003a; Craine et al. 2005; Tjoelker et al. 2005) and can vary in their slope and intercept across plant communities as a consequence of variation in climatic or edaphic parameters (Craine et al. 2005; Wright et al. 2005; Liu et al. 2010). The large magnitude and orthogonal direction of intraspecific shifts in some analogous leaf and root trait values (SLA vs. SRL, leaf vs. root DMC and P; Fig. 3) that we observed at the local scale (see also Ryser and Eek 2000) suggests a major role for plant plasticity, as opposed to species turnover, in altering these trait relationships across environmental gradients. Our findings therefore suggest that global relationships between (species mean) leaf and root traits could potentially occur but effectively disrupted by plant plastic responses to contrasting above- and belowground environmental heterogeneity occurring at multiple spatial scales (Ettema and Wardle 2002). These large partially decoupled plastic adjustments also suggest a need for many species-centred ecological theories on how plants respond to their environments (e.g. r–K strategy theory, competitive/stress-tolerator/ruderal theory, trait-based response-effect frameworks) to be adapted to account for distinct plant-environment interactions among distinct individuals of the same species and parts of the same individual.

## Conclusion

Our results highlight the potentially complex integration of multiple environmental influences by plants, stress the large potential for plastic adjustments both above and belowground and reveal that these adjustments can to some extent differ among coexisting plant species. They also suggest that while there may be broadly consistent strategies at the whole plant level among species (Grime 2001), there are also partially decoupled, finely tuned strategies between above- and belowground plant parts at the intraspecific level. These findings have a number of implications for plant community assembly, plant–soil feedbacks and ecosystem properties. For instance, they indicate that plants are capable of considerable flexibility in passing through multiple environmental filters that may exert contrasting constraints on above- and belowground organs (Díaz et al. 1998), which may potentially impact on the resilience of plant communities to environmental changes. Furthermore, our finding that trait responses to the same underlying environmental influences differed at least partially among coexisting species suggests potentially complex responses of plant communities to shifts in multiple environmental conditions. Also, given the role that plant species have as ecosystem drivers, the decoupled adjustments of leaf and root traits along gradients of environmental stresses may potentially contribute to contrasting feedbacks of litters from above- and belowground plant organs with soil properties (Wardle et al. 2004; Freschet et al. 2012). For all these reasons, our results stress the importance for ecologists to routinely consider the above- and belowground components of plants together and to recognize the major role of functional trait plasticity in driving plant responses to community assembly processes and the effects that plants have on ecosystem properties.

## Supporting Information

**Table S1.** Linear models of single and multiple environmental parameters' fit of leaf and fine root dry matter content of three sub-canopy tree species.

**Table S2.** Linear models of single and multiple environmental parameters' fit of leaf and fine root phosphorus content of three sub-canopy tree species.
