# Supplementary material for: Plasticity in above- and belowground resource acquisition traits in response to single and multiple environmental factors in three tree species
Source: Ecol Evol. 2013 Mar 7;3(4):1065–78. doi: 10.1002/ece3.520 (PMC3631414; doi:10.1002/ece3.520)
Supplement: Supplementary file 1 [file ece30003-1065-SD1.pdf]

Supporting Information

Table S1 Linear models of single and multiple environmental parameters' fit of leaf and fine root dry matter content of three sub-canopy tree species

	Leaf dry matter content (mg g^{-1})									Root dry matter content (mg g^{-1})								
	Pittosporum			*Coprosma*			*Melicytus*			*Pittosporum*			*Coprosma*			*Melicytus*		
	Sign	R^2	AIC	Sign	R^2	AIC	Sign	R^2	AIC	Sign	R^2	AIC	Sign	R^2	AIC	Sign	R^2	AIC
Burrow density	+	0.13	89.2	+	**0.43**	**90.6**	+	**0.59**	**77.1**	-	0.27	97.2	-	0.13	93.5	-	0.50	81.1
Soil *Nutrient* availability	+	0.11	89.7	+	0.10	97.0	+	0.09	86.8	-	0.64	86.9	-	**0.63**	**81.7**	-	0.58	78.8
Soil *Water* content	-	0.22	87.7	-	0.17	95.8	-	0.13	86.2	+	0.04	101.4	+	0.26	91.4	+	0.10	88.1
Light availability	+	0.13	89.3	+	0.35	92.5	+	0.41	81.7	+	0.01	101.9	-	0.06	94.6	-	0.02	89.1
Burrow + Nutrient	+ . +	0.14	91.0	+ , -	0.45	92.0	+ , -	**0.63**	**78.1**	+ , -	0.64	88.8	+ , -	**0.67**	**82.0**	- , -	0.69	77.5
Burrow + Water	+ , -	0.26	88.8	+ , -	0.49	91.2	+ , -	0.60	79.0	- , +	0.28	99.2	- , +	0.30	92.4	- , +	0.50	83.0
Burrow + Light	+ . +	0.18	90.4	+ . +	**0.54**	**89.6**	+ . +	**0.66**	**77.1**	- , +	0.40	96.3	- , -	0.14	95.4	- , +	0.58	81.0
Nutrient + Water	+ , -	0.23	89.5	+ , -	0.19	97.5	+ , -	0.15	88.0	- , -	0.68	86.8	- , +	0.65	82.8	- , -	0.60	80.4
Nutrient + Light	+ . +	0.18	90.4	+ . +	0.37	94.1	+ . +	0.41	83.6	- , +	**0.77**	**82.0**	- , +	0.63	83.7	- , +	0.60	80.3
Water + Light	- , +	0.25	89.1	- , +	0.38	93.7	- , +	0.42	83.3	+ . +	0.08	102.8	+ , -	0.26	93.3	+ , -	0.10	90.1
Burrow + Nutrient + Water	+ , - , -	0.27	90.7	+ , - , +	0.55	91.2	+ , - , -	0.65	79.2	+ , - , -	0.68	88.8	+ , - , +	0.69	83.1	- , - , -	0.71	78.5
Burrow + Nutrient + Light	+ , + , +	0.19	92.2	+ , - , +	0.57	90.7	+ , - , +	**0.70**	**77.6**	- , - , +	0.78	83.2	+ , - , -	0.67	83.8	- , - , +	**0.78**	**75.0**
Burrow + Water + Light	+ , - , +	0.28	90.5	+ , - , +	0.56	91.1	+ , - , +	0.66	79.1	- , + , +	0.43	97.6	- , + , +	0.31	94.4	- , + , +	0.59	82.6
Nutrient + Water + Light	+ , - , +	0.25	91.0	+ , - , +	0.39	95.6	+ , - , +	0.42	85.3	- , - , +	0.78	83.2	- , + , +	0.65	84.7	- , - , +	0.61	82.1

Signs of model parameters are displayed for each model. The fraction of total trait variation explained by each model is represented by regression coefficients R^2. Models with Akaike's information criterion (AIC) ≤ (lowest AIC + 1) were considered of high likelihood and indicated in bold. The number of tree populations is 15 for *Pittosporum crassifolium*, 14 for *Coprosma macrocarpa* and 12 for *Melicytus ramiflorus*.

Table S2 Linear models of single and multiple environmental parameters' fit of leaf and fine root phosphorus concentration of three sub-canopy tree species

	Leaf phosphorus content (%)									Root phosphorus content (%)								
	Pittosporum			*Coprosma*			*Melicytus*			*Pittosporum*			*Coprosma*			*Melicytus*		
	Sign	R^2	AIC	Sign	R^2	AIC	Sign	R^2	AIC	Sign	R^2	AIC	Sign	R^2	AIC	Sign	R^2	AIC
Burrow density	-	0.02	-75.7	-	0.00	-86.3	+	0.03	-47.2	-	**0.28**	**-90.9**	-	0.11	-96.1	-	0.10	-80.3
Soil *Nutrient* availability	-	0.02	-75.8	-	0.00	-86.3	+	0.33	-51.6	-	0.01	-86.1	+	0.04	-95.1	+	0.02	-79.3
Soil *Water* content	+	0.04	-76.0	+	0.07	-87.3	+	0.00	-46.8	+	0.03	-86.5	+	0.04	-95.1	+	0.02	-79.3
Light availability	-	0.00	-75.5	-	0.02	-86.5	-	0.04	-47.3	-	0.12	-87.9	-	0.10	-96.0	-	**0.19**	**-81.6**
Burrow + Nutrient	- , -	0.02	-73.8	- , -	0.00	-84.3	- , +	0.37	-50.4	- , +	**0.40**	**-91.6**	- , +	**0.40**	**-99.7**	- , +	**0.26**	**-80.7**
Burrow + Water	- , +	0.04	-74.1	+ . +	0.08	-85.4	+ . +	0.03	-45.2	- , +	0.28	-88.9	- , +	0.12	-94.3	- , +	0.10	-78.3
Burrow + Light	- , +	0.02	-73.7	+ , -	0.02	-84.5	+ , -	0.14	-46.7	- , -	0.29	-89.2	- , -	0.14	-94.7	- , -	0.20	-79.7
Nutrient + Water	- , +	0.04	-74.0	+ . +	0.08	-85.5	+ . +	0.47	-52.4	- , +	0.03	-84.5	+ . +	0.15	-94.8	+ . +	0.09	-78.2
Nutrient + Light	- , +	0.02	-73.8	+ , -	0.02	-84.5	+ , -	**0.51**	**-53.5**	+ , -	0.12	-85.9	+ , -	0.20	-95.7	+ , -	**0.30**	**-81.3**
Water + Light	+ . +	0.04	-74.0	+ , -	0.07	-85.3	- , -	0.04	-45.4	+ , -	0.12	-85.9	+ , -	0.11	-94.1	- , -	0.19	-79.6
Burrow + Nutrient + Water	- , - , +	0.04	-72.1	- , + , +	0.08	-83.5	- , + , +	0.50	-51.0	- , + , +	0.42	-90.2	- , + , +	**0.52**	**-100.7**	- , + , +	0.30	-79.4
Burrow + Nutrient + Light	- , - , +	0.02	-71.8	+ , - , -	0.02	-82.5	- , + , -	0.51	-51.5	- , + , -	0.42	-90.1	- , + , -	0.45	-98.8	- , + , -	**0.38**	**-80.8**
Burrow + Water + Light	- , + , +	0.04	-72.1	+ , + , -	0.08	-83.4	+ , - , -	0.14	-44.7	- , - , -	0.29	-87.2	- , + , -	0.14	-92.7	- , - , -	0.20	-77.8
Nutrient + Water + Light	- , + , +	0.04	-72.1	+ , + , -	0.09	-83.5	+ , + , -	**0.59**	**-53.6**	+ , + , -	0.12	-83.9	+ , + , -	0.25	-94.5	+ , + , -	0.32	-79.7

Signs of model parameters are displayed for each model. The fraction of total trait variation explained by each model is represented by regression coefficients R^2. Models with Akaike's information criterion (AIC) ≤ (lowest AIC + 1) were considered of high likelihood and indicated in bold. The number of tree populations is 15 for *Pittosporum crassifolium*, 14 for *Coprosma macrocarpa* and 12 for *Melicytus ramiflorus*.
